# Intensive procedure preferences at the end of life (EOL) in older Latino adults with end stage renal disease (ESRD) on dialysis

**DOI:** 10.1186/s12882-017-0739-7

**Published:** 2017-10-23

**Authors:** Karla Gonzalez, Jesus G. Ulloa, Gerardo Moreno, Oscar Echeverría, Keith Norris, Efrain Talamantes

**Affiliations:** 1 Department of Family Medicine, David Geffen School of Medicine at University of California, Los Angeles, Los Angeles, CA USA; 2 UCLA Family Health Center, 1920 Colorado Avenue, Santa Monica, CA 90404 USA; 3 Department of Surgery, University of California San Francisco, 513 Parnassus Ave, S-321, San Francisco, CA 94143 USA; 4 Robert Wood Johnson Foundation, Clinical Scholars Program, University of California, Los Angeles, Los Angeles, CA USA; 5 Veterans Affairs Greater Los Angeles Healthcare System, Los Angeles, CA USA; 6 UCLA David Geffen School of Medicine at University of California, 10833 Le Conte Ave # 12138, Los Angeles, CA 90095 USA; 7 Division of General Internal Medicine and Health Services Research University of California, 911 Broxton Avenue, Los Angeles, CA 90095 USA; 8 Division of General Internal Medicine, University of California, Davis School of Medicine, 4150 V Street, Suite 2400, Sacramento, CA 95817 USA

**Keywords:** Renal disease, Latinos, End of life, Advanced care planning, Dialysis, Intensive procedures

## Abstract

**Background:**

Latinos in the U.S. are almost twice as likely to progress to End Stage Renal disease (ESRD) compared to non-Latino whites. Patients with ESRD on dialysis experience high morbidity, pre-mature mortality and receive intensive procedures at the end of life (EOL). This study explores intensive procedure preferences at the EOL in older Latino adults.

**Methods:**

Seventy-three community-dwelling Spanish- and English-Speaking Latinos over the age of 60 with and without ESRD participated in this study. Those without ESRD (*n* = 47) participated in one of five focus group sessions, and those with ESRD on dialysis (*n* = 26) participated in one-on-one semi-structured interviews. Focus group and individual participants answered questions regarding intensive procedures at the EOL. Recurring themes were identified using standard qualitative content-analysis methods. Participants also completed a brief survey that included demographics, language preference, health insurance coverage, co-morbidities, Emergency Department visits and functional limitations.

**Results:**

The majority of participants were of Mexican origin with mean age of 70, and there were more female participants in the non-ESRD group, compared to the ESRD dialysis dependent group. The dialysis group reported a higher number of co-morbidities and functional limitations. Nearly 69% of those in the dialysis group reported one or more emergency department visits in the past year, compared to 38% in the non-ESRD group. Primary themes centered on 1) The acceptability of a “natural” versus “invasive” procedure 2) Cultural traditions and family involvement 3) Level of trust in physicians and autonomy in decision-making.

**Conclusion:**

Our results highlight the need for improved patient- and family-centered approaches to better understand intensive procedure preferences at the EOL in this underserved population of older adults.

## Background

Patients on dialysis are at increased risk for hospitalizations, cardiovascular events and premature mortality [1, 2]. They may also experience significant socioeconomic challenges including high poverty rates, linguistic isolation and low health literacy [3–7]. Latinos are almost twice as likely to progress to End Stage Renal Disease (ESRD), and make up a disproportionate percentage of patients on dialysis, compared to non-Latino whites [1]. Latinos with ESRD experience longer wait times before getting placed on the kidney transplant wait list [8], and are at a greater risk for hospital admissions [9]. There is growing evidence that racial and ethnic minorities also experience disparities in end of life (EOL) care [10]. National studies of Medicare beneficiaries found Latinos are more likely to be placed on mechanical ventilation, die in the hospital, and undergo interventions such as gastrostomies [11, 12]. In addition, Latinos are also less likely to participate in advanced care planning and hospice [13–17].

Individuals with advance directives (AD) report better communication about their EOL care preferences and are less likely to use intensive treatments such as feeding tubes or mechanical ventilation during their last month of life [18]. However, only 29% of older Latino patients have an AD, compared to 44% of non-Latino whites [19]. There are limited studies exploring EOL preferences and the use of intensive procedures among older Latinos with ESRD on dialysis. Improving quality, cost, and outcomes in EOL care for older racial-ethnic minorities on dialysis has significant implications. This study examines intensive procedure preferences at the end of life (EOL) in older Latino adults with and without End Stage Renal Disease (ESRD) on dialysis.

## Methods

### Study sample

The study used a convenience sample of participants with ESRD on dialysis and without ESRD, recruited through existing community partnerships. All potential participants were screened using the following inclusion criteria: age 60 and older, self-identify as Latino, Hispanic, or Chicano, ability to communicate in Spanish or English, and ability to sit for at least one hour. Patients with ESRD on dialysis were recruited from two community dialysis centers located in a predominantly Latino communities in East Los Angeles, California. Renal function status was assessed with two questions: “Have you ever been told by a physician that you have kidney failure?” and “Are you currently on dialysis?” If they answered “Yes,” to these renal function status questions they were designated as ESRD. These participants were further specified as ESRD on hemodialysis (HD) or peritoneal dialysis (PD). Individuals with ESRD on dialysis were not able to participate in focus groups, and instead participated in one-on-one semi-structured interviews to accommodate their demanding dialysis schedules.

Participants without ESRD were recruited from one senior center and one church in Los Angeles County. Renal function status was assessed with two questions: “Have you ever been told by a physician that you have kidney failure?” and “Are you currently on dialysis?” If they answered “No,” to these renal function status questions they were designated as non-ESRD and participated in one of five focus group discussions. Participants with non-ESRD recruited at both community sites were similar in terms of gender, education levels, primary language, country of origin, and SES status but those recruited from the senior center were on average older in age and had more co-morbidities. We recruited non-ESRD persons from two community sites to enhance our ability to obtain a wide range of views from non –ESRD community dwelling adults with a variation of co-morbidities.All subjects provided written consent and received a gift card for their participation. The UCLA Human Research Protection Program approved this study.

### Definitions

Intensive procedures for this study included: intubation and mechanical ventilation, tracheostomy, hemodialysis, gastrostomy tube insertion, and cardiopulmonary resuscitation.

### Focus groups

A total of five focus groups each composed of 6–10 non-ESRD participants (*n* = 47), were conducted in Spanish in private rooms at community sites, lasting approximately one hour in duration, and digitally audio recorded. The focus group facilitators (KG and JU) were trained and experienced with semi-structured interview techniques and used an interview guide containing questions and prompts. The interview guide was developed based on a review of the literature, group discussions and individual input from local experts, and formal pre-testing with community members.

During focus group discussions, facilitators described intensive procedures during EOL care (intubation and mechanical ventilation, tracheostomy, dialysis, gastrostomy tube insertion for enteral nutrition, central venous catheter for parenteral nutrition, and cardiopulmonary resuscitation). Simple images of these procedures were shown for clarity. Participants were asked to share their opinions and perceptions of intensive procedures, and to identify what they thought were the most critical factors surrounding advanced care planning (Table 1).

**Table 1:** Sample guiding questions used to facilitate groups and interview participants. Latinos without ESRD (*n*=47) and on dialysis (*n*=26)

• What have you done to prepare for end of life/death?
• Would you want these procedures done to you (images shown):
◦ intubation and mechanical ventilation
◦ tracheostomy
◦ hemodialysis^a^
◦ gastrostomy tube insertion for enteral nutrition
◦ central venous catheter for parenteral nutrition
◦ cardiopulmonary resuscitation
• When is it acceptable to do these procedures?
• When would it be wrong to do these procedures to someone?
• How did you decide what treatment you or a loved one should have?
• Who should decide what treatment you or a loved one should have?

^a^ Hemodialysis question only pertains to non-hemodialysis focus groups

### Semi-structured interviews

One-on-one semi-structured interviews were conducted with 26 ESRD participants in Spanish or English, as preferred by the participant (conducted by KG and OE). Of these semi-structured interviews, 18 participants were on HD and eight were on PD. All interviews were conducted in-person using the same guiding questions used during the focus groups (Table 1). The interviews were audio recorded, professionally transcribed, reviewed for accuracy (KG, OE) and de-identified. Recruitment ended when the full range of experiences had been exhausted and information gathered reached saturation of themes [20].

### Surveys

Prior to conducting focus groups or semi-structured interviews, all participants completed a short survey covering questions on demographics, medical care, medical conditions, and completion of advance directives.

### Analysis

Descriptive statistics were used to calculate frequencies for demographic variables. Standard qualitative content-analysis methods were used for focus group discussions and semi-structured interviews. An open coding method was initially employed by the reviewers. Transcripts were reviewed by three independent coders (KG, JU, OE) to identify broad themes and concepts within these themes. Any discrepancies in coding of the transcripts were adjudicated by an investigator on the team (ET). Based on independent analysis, a comprehensive code book listing all codes generated was developed and used by the team in the final round of review.

## Results

### Quantitative analysis

Participants were on average 70 years old with a wider standard deviation for the ESRD participants compared to non-ESRD participants. A larger percentage of participants in our non-ESRD group were female, compared to the ESRD participants. Most participants identified Mexico as their country of origin. Nearly 60% of all participants had completed less than eighth grade level of primary education. A higher percentage of ESRD participants (61.5%) self-rated their health as fair or poor, as compared to the non-ESRD participants (46.8%). ESRD participants were observed to have a higher number of co-morbidities, and a higher proportion reporting a functional limitation (Table 2).

**Table 2:** Participant survey characteristics collected in 2016 among Latino non-ESRD and Dialysis patients from Los Angeles County

Characteristic	Non-ESRD *n* = 47 (100%)	Dialysis^a^ *n* = 26 (100%)
Age, mean (SD)	70.5 (8.1)	70.5 (9.2)
Female, n (%)	32 (68.1)	12 (46.2)
**Country of origin, n (%)**		
United States (US)	7 (14.9)	3 (11.6)
Mexico	37 (78.7)	18 (69.2)
Central America	3 (6.4)	5 (19.2)
**Years lived in the US if born elsewhere, n (%)**		
≥ 30 years	31 (66)	15 (57.7)
Prefer communicating in Spanish, n (%)	47 (100)	24 (92.3)
Married, n (%)	19 (40.4)	15 (57.7)
**Education level completed, n (%)**		
Less than primary grade school (≤8th)	28 (59.6)	15 (57.7)
Secondary grade school (9th – 12th)	14 (29.8)	9 (34.6)
College or higher	5 (10.6)	2 (7.7)
**Annual Income, n (%)**		
< $15,000	21 (44.7)	16 (61.5)
≥ $15,000	18 (38.3)	7 (27)
No answer or do not know	8 (17.0)	3 (11.5)
**Health Insurance, n (%)**		
Medicare-Medicaid	19 (40.4)	10 (38.5)
Medicare	8 (17)	5 (19.2)
Medicaid	8 (17)	7 (26.9)
Kaiser or other HMO	4 (8.5)	2 (7.7)
Uninsured	5 (10.7)	0 (0)
No answer or do not know	3 (6.4)	2 (7.7)
**Self-rated health, n (%)**		
Excellent/Very Good/Good	25 (53.2)	10 (38.5)
Fair/Poor	22 (46.8)	16 (61.5)
**Co-morbidities categorical, n (%)**		
≤ 2	9 (19.1)	0 (0)
≥ 3	38 (80.9)	26 (100)
One or more ED visits in past year,^b^ n (%)	18 (38.3)	18 (69.2)
Have one or more functional limitations,^c^ n (%)	18 (38.3)	18 (69.2)

*US* United States, *HMO* health maintenance organization, *ED* emergency department

^a^ESRD on dialysis group includes patients on hemodialysis or peritoneal dialysis

^b^Self-reported ED visits

^c^ Self-reported functional limitation

With regards to EOL care preferences, more than 90% of participants in both groups had identified someone they would trust with medical power of attorney (POA). For the non-ESRD participants, the most common person identified was a child or grandchild (59.6%), while ESRD participants more commonly identified their spouse or partner (57.7%). Nearly two thirds of non-ESRD participants had discussed EOL care with a trusted person, while less than half of ESRD participants had discussed EOL care with a trusted person. Among both ESRD and non-ESRD participants, less than 25% of had written instructions for EOL care or had filed a POA (Table 3).

**Table 3:** Advanced care planning and religiosity among Latinos without ESRD (*n* = 47) and with dialysis (*n* = 26)

Characteristic	Non-ESRD *n* = 47 (100%)	Dialysis^a^ *n* = 26 (100%)
Have identified person they would trust with medical power of attorney (POA), n (%)	43 (91.5)	24 (92.3)
Spouse or Partner	11 (23.4)	15 (57.7)
Child or Grandchild	28 (59.6)	8 (30.8)
Other^b^	8 (17)	3 (11.5)
Discussed end-of-life-care with trusted person, n (%)	34 (72.3)	11 (42.3)
Have filed a POA, n (%)	11 (23.4)	5 (19.2)
Have left written instructions, n (%)	12 (25.5)	5 (19.2)
**Believes family and/or friends would have problems following their wishes, n (%)**		
Yes	3 (6.4)	2 (7.7)
No	38 (80.8)	24 (92.3)
Don’t Know/NA	6 (12.8)	0 (0)
**Believes personal physician would have problems following their wishes, n (%)**		
Yes	0 (0)	12 (46.2)
No	42 (89.4)	12 (46.2)
Don’t Know/NA	5 (10.6)	2 (7.6)
**How strongly religious are you? n (%)**		
Very strong	21 (44.7)	11 (42.3)
Somewhat strong	20 (42.6)	11 (42.3)
Not very/Not at all strong	6 (12.7)	4 (15.4)
**Religion is important in my everyday life, n (%)**		
Strongly agree	36 (76.6)	18 (69.2)
Somewhat agree	8 (17)	4 (15.4)
Somewhat disagree/Strongly disagree	3 (6.4)	4 (15.4)

*POA* power of attorney, *NA* not applicable

^a^ESRD on dialysis group includes patients on hemodialysis or peritoneal dialysis

^b^Other – family member, friend, physician, medical professional, or other

### Qualitative analysis

Focus group sessions lasted approximately one hour to one hour and 15 min. Individual interviews lasted between 40 min to one hour. We identified three broad overarching themes: 1) The acceptability of a “natural” versus “invasive” procedure, 2) Cultural traditions and family involvement, and 3) Level of trust in physicians and autonomy in decision-making.

#### The acceptability of a “natural” versus “invasive” procedure

Both ESRD and non-ESRD participants determined if a procedure was acceptable by first assessing if it was “natural,” versus “invasive.” Invasive procedures were described as “going against what the body wanted to naturally do” and “forcing the body away” from the dying process. For example, both ESRD and non-ESRD participants agreed that intubation, mechanical ventilation, and tracheostomy can be invasive. One participant shared “If my body cannot survive on its own without invasive means like machines, then just let me go.”

In contrast, “natural” procedures were described as those that could keep patients alive without a significant amount of physical and emotional suffering by assisting the body in its innate healing process. Among the non-ESRD participants, hemo- and peritoneal dialysis, and kidney transplants were considered natural. A non-ESRD participant stated, “I’m ok with dialysis because I see that patients on dialysis can live a normal life.” Similarly, participants described enteral or parenteral nutrition as natural, because it could help someone recover from acute illness. One non-ESRD participant describes a situation involving a relative when, “They had to switch from a [NG tube] to [G-tube], she was still strong, but just needed more nutrients to regain her strength while very ill.” One ESRD participant shares, “My wife had a hard time swallowing, but other than that she was fine, so of course they put it because that’s how they had to feed her.”

Natural procedures are further characterized by their outcomes, which was different for non-ESRD and ESRD participants. Some of the non-ESRD participants described dialysis as an enhancement of health rather than a life-sustaining intervention. A non-ESRD patient stated that his family member, “was already 92 and his body looks fine, [but] on the days when he gets dialysis he looks great!” Other non-ESRD participants made similar comments about dialysis. In contrast, ESRD participants described the outcomes of dialysis more concretely and were able to identify it as a life-sustaining process. HD participants described dialysis as “doing what the kidneys should normally be doing.” Participants on PD were even more specific stating “it helps remove the phosphorus which makes me itch…it removes the water in my lungs when I can’t breathe.” Both PD and HD ESRD participants expressed that without dialysis they would die.

A difference among ESRD participants, was the desire to continue more intensive procedures regardless of risks or limitations. For example, some PD participants shared that it would be worthwhile to continue all forms of treatment even if it would only add a few more days of life. “I told my children, please take me to the hospital so they can treat me, and spend your money on me, even if it only gives me a few more days.” However, both ESRD and non-ESRD participants described not wanting intensive procedures if they were diagnosed with a terminal cancer diagnosis, in which case most participants agreed that all procedures were futile, increased suffering, and should be stopped.

#### Cultural traditions and family involvement

The majority of participants acknowledged that they had not given much or any thought about their preferences regarding intensive procedures. They did however, report other topics that were important in thinking about their death. For example, both non-ESRD and ESRD participants felt that preparing for a traditional burial was important. They felt that adhering to cultural traditions could minimize emotional and economic burden on their family. Several participants stated that it is customary to save money to cover expenses for their funeral, as well as discuss details about the funeral with their children. Many stated this was important to them and they were trying to do just that, even though none of them were actively passing away. They believed this would avoid major conflicts among family members during EOL and after their death. One participant shared “I already have a burial plot at the cemetery, near my husband, I have money for each of my children, I have asked my children to bring the mariachi, and the songs I wish for them to play, and I told them how to divide the land I have in Mexico. I’m ready, and this way, I avoid discord between my children.”

Both ESRD and non-ESRD participants also believed that casual conversations regarding their EOL preferences with certain family members were sufficient preparation for EOL. One participant shared “I discussed this issue with my daughters, not my sons because I cannot count on them. I have made it clear to my daughters, and they understand that under no circumstances do I want invasive life, no tubes, just let me die natural.” Participants did not offer their views on the utility of written forms of ADs or conversations with MDs to further guide end of life care, if family was not available. When probed on this, most participants verbalized that having a written AD or discussing medical care wishes with MDs was probably useful, even though they themselves had not done it.

A distinction between the non-ESRD group and ESRD group, was the level of involvement of family in EOL decision-making. Among the non-ESRD participants, many wanted family present during EOL discussions, but expressed a desire for making their own medical decisions when possible. “It’s always better if you don’t have to leave the hard decisions up to them.” Several participants even knew that there were ways to document preferences for EOL care. “There is a lot of information out there, and a form called five wishes, and you can indicate what you want and it prevents your family from having to make those decisions.”

In contrast, the ESRD participants saw procedural interventions as immediate events that cannot, and should not be planned for, even if they had already experienced a severe life-threatening hospitalization. One participant shared “when I got sick and they had to intubate me, it was so sudden and I didn’t have time to even think about it. So you never know when it’s going to happen to you so why even plan for it?” ESRD participants more often expressed that they believed their children (less often their spouses), would and should be responsible for making those decisions. Knowledge about written ADs among ESRD patients was secondary to their inherent exposure to healthcare settings due to their disease. One ESRD patient stated, “I did fill that form out…they told me I had to fill it out if I wanted to be on dialysis, but before that I didn’t even know about it.”

Both ESRD and non-ESRD participants used spirituality and religion in determining when life-sustaining procedures should be stopped. Often these difficult discussions included the guidance of a spiritual or religious leader. A non-ESRD participant stated, “Father [name] told me, if [patient name] was still with life, he would tell you himself, ‘let me go, I am suffering too much.’ It would have been wrong to keep him going.” Several ESRD participants stated that “you cannot go against what God has already decided. God was ultimately the one who decided when your life was over.” A Non-ESRD participant added “If God thinks it is their time, then none of what you do will help…you will only make them suffer.”

#### Level of trust in physicians and autonomy in decision-making

An apparent distinction between ESRD and non-ESRD participants was the trust they had in their physicians. Among the non-ESRD group participants, there was a general agreement that one should not take physicians advice at face value. One non-ESRD participant shared “a doctor told me there was no hope for me, but then the cardiologist came and said he would send me to a specialist who could heal me so I went there.” Several non-ESRD participants expressed frank mistrust of physicians and wondered if they were not guided by alternative [monetary] motives “I’m not saying all of them, but some physicians are like vultures, just waiting until you get sick so they can take and take.” Another ESRD participant shared of someone he knew, “the man no longer spoke, and was no longer conscious of himself and I asked why, why they had him on life support, and you know why? It was so they can keep billing.” Others in the non-ESRD group stated that when they did not receive enough information, or conflicting information from different physicians, they were more likely to keep interventions going on longer. A participant shared that a doctor “didn’t really give me options. He didn’t explain well. He said he *had* to put the feeding tube [G-Tube], or he would die in 4 days. I had no idea what to do and I looked to God, I was alone without my children so I said “Go ahead and put it!” And he lasted six months after that.” In contrast patients who received a consistent message from the entire team of physicians felt comfortable in withdrawing care.

In general, ESRD group participants appeared to have a more trusting relationship with their physician. “They saved my life once, so they know what they are talking about. We just do whatever the doctor says is best.” When asked about intensive procedures, they generally took a more passive role, looking to their physician for answers rather than asking questions. “They didn’t ask me if I wanted hemodialysis or peritoneal dialysis, they just said this one was better because I was having too many infections on my arm with the other one.” PD patients appeared to have a particularly trusting relationship with their physician. As one person stated, “I trust him because he told me about the process and what it did to my body, and that eventually I was going to need a transplant. No one else had told me all the options and now I’m definitely going to try to get on that list!” Additionally, HD and PD patients were much more inclined to let doctors decide treatment at EOL as they felt they did not have enough knowledge to contribute to the decision-making. One participant shared that his “doctor decided that it was time for dialysis, they said it was that or I would die, so I went with that.” Another PD ESRD participant stated, “When my son in law was beat up, he was practically dead. His brain didn’t work. They kept him alive on the machine. My daughter just figured that the doctors thought he just needed more time on the machine. Then after a long time, they told her it was time to take him off. I guess it didn’t work.”

Among non-ESRD and ESRD participants, there was a common perception regarding Cardio Pulmonary Resuscitation (CPR). CPR was thought of as a “duty” that physicians must carry out. A non-ESRD participant said, “They at least have to try the chest compressions. That’s part of their job so it’s not really up to us.” An ESRD participant stated “Well, they have to do that because if they don’t they’ll get sued. People will say that they didn’t try their best to save them.”

## Discussion

This exploratory study found that patients with ESRD on dialysis prefer the continuation of intensive procedures, even when “invasive.” While most of the participants have discussed EOL preferences with family members, less than 25% of had written instructions for EOL care or had filed a POA. Intensive procedure preference was described as relying on the distinction between “natural,” versus “invasive.” Clarifying this distinction may assist clinicians in explaining the purpose of an intensive procedure to support patient-centered and shared decision-making. Patients with ESRD have often undergone various life-sustaining intensive procedures, and may have a higher acceptability for intensive procedures or dying in the hospital, as previously described [11, 12].

Cultural traditions and family involvement also played a significant role in intensive procedure preferences. This study provides new results relevant to older Latinos with and without ESRD on dialysis. Our study describes religious leaders as significant in in determining when life-sustaining procedures should be stopped. Including religious leaders and family members in discussions regarding intensive procedure decisions may further ensure a patient-centered approach to EOL care. These findings further support the importance of religion and religious leaders when making difficult EOL care decisions as described in studies of multiethnic populations with advanced cancer and near death [17, 21].

This study also found Latinos prepare for death by buying burial plots, and having verbal conversations with their children about burial traditions. This highlights that this population does plan and discuss their EOL wishes, albeit not necessarily via written documentation. These already-accepted preparations and conversations can serve as opportunities for more structured EOL discussions. Future interventions may focus on modifying these already existing conversations to include intensive procedures preference and other important EOL care planning.

This study also highlights the importance of a family-centered approach to EOL care when caring for older Latinos [22]. In this study ESRD patients primarily deferred EOL care decision-making to their children, however they did not discuss intensive procedure preferences. In contrast, non-ESRD patients preferred to make their own EOL care decisions to avoid leaving the hard decisions to family members. This distinction can be useful in tailoring the family-centered conversation when dealing with each of these populations.

Another major finding of this study was the trust participants expressed when discussing EOL care with a physician. The level of trust patients had in their physician varied between the ESRD and non-ESRD group. ESRD patients were more trusting of their physicians when deciding on intensive procedures. Patients who have undergone life-saving procedures like dialysis with good outcomes, may find it easier to trust a physician when deciding on getting an intensive procedure. In contrast, the non-ESRD group preferred to actively make their own decisions regarding intensive procedures. For the non-ESRD group these findings contradict prior research that describes Latinos taking a more passive role [23].

Our study results expand the relevant literature in other important areas. This is the first study to explore the reasons for intensive procedure preferences among older Latino adults with ESRD on dialysis. Furthermore, our sample was unique in that the majority of participants were older Latinas with a primary education who identified Mexico as their country of origin. In research, this is considered a hard to reach and understudied population. Previous similar studies did not focus on those with ESRD but instead explored EOL care preferences among persons with cancer, and like those studies we found that Latinos with ESRD on dialysis are more likely to accept intensive procedures.

This study has several limitations. The initial design of this study was to hold focus groups for both ESRD and non-ESRD participants. ESRD participants were only able to participate in individual interviews and not focus groups because of demanding dialysis schedules. Focus groups and individual interviews can result in different responses, even when the same leading questions are used. Our sample consisted of a convenience sample of community dwelling older Latino adults which may limit the generalizability of our findings. We recruited participants from Los Angeles County dialysis and community sites and our results may not generalize to other geographic areas or all Latino populations. The majority of the participants preferred to communicate in Spanish, which may omit findings that were revealed by focus groups and interviews conducted in English. Though our aim was to obtain a sample of participants with ESRD on dialysis and one without ESRD not on dialysis, we cannot ensure that the participants designated non-ESRD do not have chronic kidney disease. We did not collect demographic data, such as race-ethnicity, on the healthcare providers of participants or dialysis center patients. The interpretation of the qualitative data is subject to bias from investigators. We potentially limited bias by independently coding transcripts and assigning thematic titles that were then discussed and collapsed through adjudication. This study was hypothesis generating and more research is needed to test hypotheses that may help improve Latino patients’ understanding of procedures during EOL care. Finally, not addressed in this study are the structural factors that hinder improvement in EOL care. For example, many patients noted that their physicians had never outlined the options available or not available to them. The lack of conversations about prognosis and appropriate versus inappropriate interventions was not addressed in this study.

## Conclusions

In summary, this is the first study to investigate preferences of intensive procedures in this specific population of older Latinos with ESRD on dialysis. This study found some differences in intensive procedures preferences among Latinos with ESRD on dialysis and those without ESRD. It also provides insight into the possible framework this group may use to determine the acceptability of a procedure. The study reveals that while Latinos may not be completing AD forms, they do have other traditional and important ways to communicate their general preferences regarding EOL care with their children. It also explores the impact of trust or mistrust in physicians, on intensive procedure preference. These findings among the ESRD and non-ESRD participants highlight the need for different approaches to discussions regarding intensive procedure preference and EOL care that may vary based on severity of illness or prior experience with procedures [23, 24].

Our results have clinical implications and provide clinicians with important themes that may improve EOL care discussions with Latino patients. Use of these themes may help generate more effective EOL care discussions in older Latino adults, specifically those with kidney disease, and is an important step toward end of life care planning and reducing EOL care disparities in this minority population.

## Abbreviations

AD: Advance Directive(s)
EOL: End of life
ESRD: End stage renal disease
ESRD: Group-participants who indicated they have kidney disease, are on dialysis, and participated in one-on-one interviews
HD: Hemodialysis
Non-ESRD: Participants who indicated they have never been told they have kidney failure, are not on dialysis, and participated in focus group discussions
PD: Peritoneal dialysis
POA: Power of attorney
